# Interventions to Promote Positive Mental Health in Family Caregivers: a Systematic Review

**DOI:** 10.1590/0034-7167-2024-0622

**Published:** 2026-07-31

**Authors:** Sandra Marisa Barbosa de Alpuim-Gonçalves, Vânia Lídia da Silva Soares, Ana Isabel Fernandes Querido, Catarina Cardoso Cardoso Tomás, Carmen Maria da Silva Maciel Andrade

**Affiliations:** I ISAVE - Higher Institute of Health. Amares, Braga, Portugal; II Universidade do Porto. Porto, Portugal; III Instituto Politécnico de Leiria. Leiria, Portugal; IV Universidade dos Açores. Ponta Delgada, Portugal

**Keywords:** Evidence-Based Nursing, Family Caregivers, Mental Health, Health Literacy, Systematic Review., Enfermagem Baseada em Evidências, Cuidadores Familiares, Saúde Mental, Letramento em Saúde, Bem-Estar Psicológico., Enfermería Basada en la Evidencia, Cuidadores Familiares, Salud Mental, Alfabetización en Salud, Bienestar Psicológico.

## Abstract

**Objectives:**

to identify interventions aimed at improving the mental health of family caregivers, whether or not focused on health literacy and health gains.

**Methods:**

this systematic review was conducted in accordance with the Joanna Briggs Institute guidelines and involved searches in nine databases. Primary studies published in English, Spanish, and Portuguese were included if they developed, tested, or implemented programmes designed to promote positive mental health among caregivers aged 18 years and older.

**Results:**

seven original studies were included, comprising randomised controlled trials, quasi-experimental studies, mixed-methods studies, and pilot studies conducted across different countries. The interventions ranged from home visits and problem-solving techniques to online psychoeducational courses and caregiver training programmes. Significant improvements were observed in depression, anxiety, caregiver burden, and positive mental health, alongside a significant increase in caregivers’ skills and knowledge.

**Conclusions:**

this review demonstrates that educational and psychosocial approaches contribute positively to improving the mental health of informal caregivers.

## RESUMO

**Objetivos:**

identificar intervenções que melhorem a saúde mental de cuidadores familiares, seja com foco em alfabetização em saúde ou ganhos de saúde.

**Métodos:**

esta revisão sistemática seguiu as diretrizes do Joanna Briggs Institute e selecionou estudos de nove bancos de dados. Foram incluídos estudos primários publicados em inglês, espanhol e português que desenvolveram, testaram ou implementaram programas voltados a promover a saúde mental positiva entre cuidadores com 18 anos ou mais.

**Resultados:**

incluíram-se sete estudos originais, compreendendo ensaios clínicos randomizados, estudos quase experimentais, estudos de métodos mistos e estudos-piloto de diferentes países. As intervenções variaram de visitas domiciliares e técnicas de resolução de problemas a cursos online de psicoeducação e treinamentos para cuidadores. Os resultados indicaram melhorias significativas na depressão, na ansiedade, na carga do cuidador e na saúde mental positiva, além de um aumento relevante nas habilidades e conhecimentos dos cuidadores.

**Conclusões:**

esta revisão demonstra que as abordagens educacionais e psicossociais contribuem positivamente para a melhoria da saúde mental de cuidadores informais.

## RESUMEN

**Objetivos:**

detectar intervenciones que buscan mejorar la salud mental de los cuidadores familiares, ya sea que se enfoquen en la alfabetización en salud o en otras mejoras en la salud.

**Métodos:**

esta revisión sistemática se realizó siguiendo las directrices del Instituto Joanna Briggs, realizando búsquedas en nueve bases de datos. Se incluyeron estudios primarios publicados en inglés, español y portugués que desarrollaron, probaron o implementaron programas destinados a promover la salud mental positiva en cuidadores mayores de 18 años.

**Resultados:**

se incluyeron siete estudios originales, entre ellos ensayos controlados aleatorios, estudios cuasiexperimentales, de métodos mixtos y pilotos, realizados en diversos países. Las intervenciones variaron desde visitas domiciliarias y técnicas de resolución de problemas hasta cursos psicoeducativos en línea y programas de capacitación para cuidadores. Se evidenciaron mejoras significativas en la depresión, la ansiedad, la carga de los cuidadores y la salud mental positiva, además de un aumento notable en habilidades y conocimientos.

**Conclusiones:**

la revisión confirma que los enfoques educativos y psicosociales brindan beneficios significativos para la salud mental de los cuidadores informales.

## INTRODUCTION

The number and proportion of adults with noncommunicable diseases (NCDs) and aged 60 years and older in the population is increasing^(1,2)^. These adults usually need complex care which is considered a global health problem. Due to the personal suffering and financial burden it generates, it has a strong impact on the lives of communities and health systems. Most of these adults with chronic conditions or disabilities live in their homes and the help is provide by informal caregivers, usually a relative, partner or friend. The informal caregivers provide unpaid help with a variety of aspects of self-care, and they do not receive specific training for the care they provide or information on how to cope with the caregiving burden^(3)^.

Caregiving is a time-consuming process that requires time for the caregiver to attend to their own emotional, psychological, physical, social, leisure, or spiritual needs^(4)^. The negative impacts of caregiving are unequivocal and vary among caregivers^(5)^.

The WHO defines health as “a state of complete physical, mental and social well-being and not merely the absence of disease or infirmity”^(6)^. Thus, the highest standard of health for caregivers includes well-being and mental health^(7,8)^.

Prevention and promotion of mental health are essential to reduce the increasing burden of mental disorders on the individual and society. Strategies for mental health promotion are related to positive mental health and not to mental ill health^(7,8)^.

Positive mental health has been highlighted in the literature as an essential part of the overall health. The term ‘positive’ refers to promoting actions to strengthen and enhance mental health in general.

Health literacy represents a fundamental tool for health promotion, mainly in training people to achieve their life projects, which justifies the planning of interventions to achieve health gains. In the context of mental health, health literacy can be defined as a person’s capability to obtain and maintain positive mental health, understand mental disorders and treatments, reduce the stigma related to mental disorders and improve the effectiveness of help-seeking interventions^(9)^.

The literature shows that some interventions have been designed to improve positive mental health in informal caregivers, whether using health literacy strategies or not.

The preliminary search in the JBI Database of Systematic Reviews and Implementations Reports, the Cochrane Database of Systematic Reviews, CINAHL (via EBSCO), the Database of Systematic Reviews, MEDLINE (via PubMed), Scielo and Prospero did not find literature reviews (published or to be developed) in the study area.

## OBJECTIVES

To identify the interventions designed to improve mental health in caregivers, focused or not on health literacy and their health gains.

## METHODS

The systematic review was developed according to the Joanna Briggs Institute systematic reviews of measurement properties^(10)^ to answer the following review questions: “What are the interventions designed to improve positive mental health in family caregivers”; What are interventions focused on health literacy that improve positive mental health in family caregivers? Are there health gains of health literacy interventions to improve positive mental health in family caregivers? The study protocol was registered in PROSPERO (registration number CRD42022343148, available at https://www.crd.york.ac.uk/PROSPERO/view/CRD42022343148).

### Search strategy

A systematic review was conducted through databases and scientific repositories and was limited from January 1, 2017 to March 8, 2022.

According to the JBI recommendations, the search strategy included an initial search for studies published in the PubMed, CINAHL, and Scielo databases using the keywords “mental health” OR “caregivers” AND “Program*” OR “families”, which aim was to identify in titles and abstracts of the studies, the indexed and free text terms most frequently used in this area of study.

A second search was conducted with the indexed search terms and keywords, adapted and individualized to the following electronic databases: MEDLINE^®^ (Medical Literature Analysis and Retrieval System Online), CINAHL^®^ (Cumulative Index to Nursing and Allied Health Literature), Scopus^®^, Cochrane Library, SciELO ( Scientific Electronic Library Online), BVS (Virtual Health Library), PsycINFO (American Psychological Association, access via EBSCOhost Web), JBI Evidence Synthesis and Web of Science. The grey literature was mapped using the databases RCAAP (Scientific Repository of Open Access of Portugal) and OpenGrey. This research was carried out from March to June 2022.

The search strategy used for Scopus is provided in Chart 1.

**Chart 1:** Search strategy

Research Strategy Scopus (June 30, 2022)		Results
S1	“mental health” OR “mental hygiene” OR “positive mental health” OR “positive psychology” OR “psychology, positive” OR “well-being” OR “mental well-being” OR “life satisfaction” OR “subjective well-being” OR “psychological well-being” OR “social well-being” OR “personal satisfaction”	(N=242,614)
S2	famil^*^ OR Caregiver^*^ OR “Informal caregiver^*^” OR Carer^*^ OR “Spouse caregiver^*^”	(n=595,860)
S3	“Mental Health Literacy” OR “Health literacy”	(n=11,851)
S4	Intervention^*^ OR Program^*^ OR Promotion^*^ OR Impact^*^ OR Program development OR Home Health Nursing OR “Evidence-Based Nursing”	(n=9,640)
S5	S1 AND S2 AND S3 AND S4	(n=4)

A manual search was performed with the Google Scholar web search using the keywords (“positive mental health” AND “program development” OR “Mental Health Literacy”). Studies identified were screened manually to check all references and to identify potentially relevant studies. When the studies’ methodology did not properly describe the instruments, the respective validation studies were identified as a source of additional psychometric information, regardless of the year of publication. Two independent reviewers performed the search.

During the initial phases of our study, a comprehensive search of various databases was conducted to gather relevant literature and data. However, the development of the study took longer than initially expected, which justified the performance of a new search in the CINAHL^®^, PubMed, Scopus^®^, SciELO and PsycINFO databases to ensure the accuracy, relevance and completeness of the results of our research.

This new search included studies from June 2022 to May 2024 and ensured that our research remains current, comprehensive, and of the highest quality, thereby enhancing our study’s overall contribution to the field.

### Study eligibility criteria

Studies were eligible if they included any program developed, tested, or used after 2017 to promote the positive mental health of caregivers over 18 years of age, including published and unpublished primary studies, regardless of the country or setting.

Studies published in English, Portuguese, and Spanish were included, based on the reviewers’ level of linguistic proficiency, ensuring greater rigour in the selection of evidence and data extraction.

We excluded studies whose methodology was not properly described and/or the authors provided no additional information about the intervention, as well as systematic reviews and qualitative research.

The inclusion process exceptionally added an article outside the defined period, justified by highly relevant information or results that were not covered in other sources within the specified period^(11)^. Its inclusion was essential to provide a comprehensive and accurate understanding of the topic, contributing significantly to our field of research and its authorship being from recognized experts. It is a program based on the Problem-Solving technique, one of the factors associated with the Multifactorial Model of Positive Mental Health presented by Teresa Lluch^(12)^.

### Study Selection

Two reviewers performed this process which is presented and organized using the PRISMA 2020 flow diagram^(13)^.

Articles were screened for inclusion using Endnote X9^®^ Software reference management software (Clarivate Analytics, PA, USA) and duplicate references were identified and removed. Two authors independently screened the titles and abstracts. The same two authors assessed the potentially relevant full-text information. Disagreements between the two reviewers at any stage of this process were resolved by consensus or through analysis and discussion by a third reviewer.

In a second phase, the authors read the articles in full, evaluating them individually. By consensus, those that were considered relevant were introduced into a methodological quality verification grid, according to the JBI criteria^(10)^.

### Assessment of methodological quality

Two independent researchers assessed all eligible studies using the JBI Critical Appraisal Checklist for Randomized Controlled Trials^(14)^. Any researcher disagreement was resolved through discussion, or with a third reviewer. The quality of each eligible study and the minimum score for the inclusion of each original study in this systematic review were based on criteria established by the authors. Therefore, the inclusion of each study is based on a pre-determined low-quality score. The quality score of each study was based on the following cut-offs: 0-3 was considered a very low-quality score; 4-6 was considered a low-quality score; 7-9 was considered a moderate-quality score; and 10-11 was considered a high-quality score. The authors pre-established a minimum score of 4 as a criterion for inclusion in this systematic review.

### Evidence synthesis

Evidence synthesis was conducted according to the JBI guidelines^(10)^. A grid was used where the studies were introduced and coded and where each reviewer substantiated the reason for excluding the remaining articles. The extraction of data from each study was carried out using a grid where the following analysis criteria were introduced: author, publication date, country, program name, goals, target population, number of sessions, duration of sessions, intervenor, strategies used, mode of intervention, instruments used in the measurement, program outcomes and literacy domains of the programs promoting positive mental health literacy of the described family caregivers.

## RESULTS

The initial search strategy identified 820 studies. After removing duplicates and records marked as ineligible by automation tools, 556 studies were screened by the relevance of their titles and abstracts. To identify potentially relevant studies, we performed a manual search, which yielded 15 studies. The full text of the remaining 53 studies was assessed for eligibility, and four original studies were included in this review.

A second search was developed between June 2022 and May 2024 with the aforementioned methodology. It was conducted, allowing us to identify 6,834 studies, from which 558 duplicates were removed, leaving 6,276 for analysis based on the relevance of their titles and abstracts. A total of six studies were assessed for eligibility by full test analysis.

**Figure 1:**
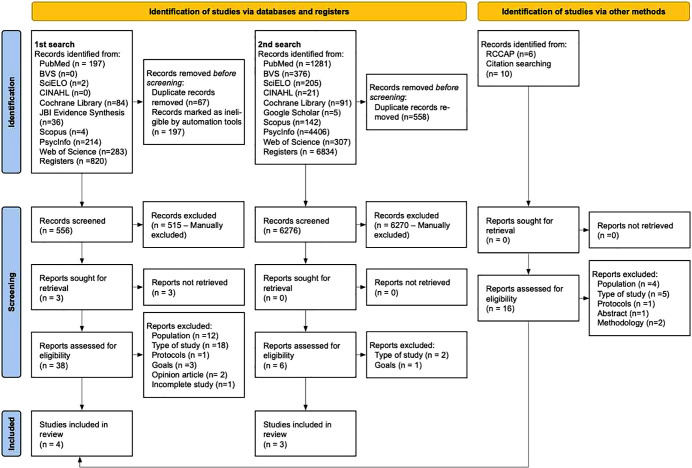
PRISMA 2020 flow diagram for new systematic reviews^(13)^

Overall, seven studies were included in this review, with four originating from the first search and three from the second. The PRISMA 2020 flow diagram (Figure 1) presents the study selection process for both searches conducted in this systematic review.

### Quality appraisal

All studies assessed were included in this review with agreement among the researchers. The 13 quality appraisal criteria listed in the JBI critical appraisal checklist results, for each of the seven studies, are presented in Supplementary File 1.

Two of the seven reviews were rated as high quality^(15,16)^, four as moderate^(11,17-19)^, and one low quality^(20)^. Four of the 13 criteria (30,8%) were met by all studies. Two criteria (item five and six) were not met in any study included.

The minimum number of criteria was four, and the maximum was 11. No review was excluded based on methodological quality criteria.

### Characteristics of included studies

In this systematic review seven original studies were included. Regarding study designs, three studies described the randomized controlled trial (RCT)^(11,15,16)^. One paper described a quasi-experimental study^(18)^, two mixed methods studies^(19,20)^, and one pilot study^(17)^. The year of publication of the studies ranged between 2013 and 2024. Two of the seven were developed in Spain^(11,15)^, one in Singapore^(17)^ , one in Iran^(18)^, and one in Finland^(20)^, one in Australia^(16)^ and one in Germany^(19)^.

The target population was adult (over 18 years) caregivers. Six reviews included only caregivers, and one included caregiver-patient dyads^(16)^. More detailed information from each study was summarized in Chart 2.

**Chart 2:** Summary of findings from the included studies

Author/ year	Study design	Intervention	Instruments	Outcomes assessed	Domains of health literacy assessed	Results
**Jiang et al. 2024^(17)^**	Pilot study design	Programme (C2C)	Dmographic questionnaires and evaluation questionnaires	Knowledge and skills development, self-care ability, trainer engagement and training content	Not reported	Results showed that their caregiving knowledge, skills and selfcare ability were improved and the positive effects were maintained above baseline over the course of C2C and at 2-month follow-up Participants gained significant support from other caregivers and healthcare professionals
**Mousaei et al., 2023** ^(18)^	Quasi-experimental study	Family-centered empowerment model (FCEM)	Demographic information questionnaire and Zarit caregiver burden inventory, including 22 questions about the burden imposed by a caring a patient on the caregiver and the responses are based on Likert scale (never = 0, rarely = 1, sometimes = 2, often = 3, and always = 4).	Level of care burden	Not reported	Intervention was effective in the changes of care burden scores (p < 0.036) and the interaction between time and treatment was not significant (p < 0.053). No statistically significant difference between the two groups (intervention and control) in terms of the care burden mean scores (p < 0.225)
**Laine et al., 2021^(20)^**	Parallel mixed methods study design	Web-based psychoeducation course	Assessment of participants activity on the learning platform. Number of caregivers visiting every module and the number of finalized module tasks (calculated manually) Written feedback about course and the website were asked to participants Sociodemographic Information	Caregivers’ engagement Feedback about the course	Not reported	The web-based psychoeducation course for caregivers seems to be especially suitable for those who have little experience as a caregiver. Less than two-thirds (18/30, 60%) completed the course. Feedback on the course varied: over half (10/17, 59%) of the caregivers considered the content to be very good or good, about half (9/17, 53%) considered the website layout to be good, only 6% (1/17) felt that the usability of the website was poor, and no one felt that it was very poor.
**Ferré-Grau et al., 2021^(15)^**	RCT	TIVA App	Positive Mental Health Questionnaire (PMHQ) Zarit Caregiver Burden Interview (ZBI-7).	Caregivers’ outcomes: Positive mental Health; Caregiver burden.	Not reported	**Positive mental health:** Personal factor (factor 1 of the PMHQ): showed a significant difference between the groups, but it was not clinically relevant (0.96; p=0.03). IG obtained a higher mean change for the overall PMHQ score (mean change between groups:1.40; p=0.24); after the third month of the intervention showed an increment of PMHQ scores. The mean difference of change in the PMHQ score showed a significant difference between the groups (11.43; p<.001; d=0.82); were reported significant changes in 5 of the 6 factors, especially: (F 5) Problem solving and self-actualization (5.69; p<0.001; d=0.71), (F2) Prosocial attitude (2.47; p<0.001; d=1.18), and ( F3)Self-control (0.76; P=.03; d=0.50). **Caregiver burden:** results showed a decrease in caregiver burden in the intervention group.
**Krieger et al., 2020^(19)^**	Mix Study	Via outreach counselling	Two instruments were developed using the inputs of a combined stakeholder and risk analysis A semi-structured questionnaire measured health literacy and psychosocial health using 21 items on a five-point Likert scale (1 ‘very negative’ to 5 ‘very positive’). Health literacy was assessed using Freebody and Luke’s (1990) framework: Functional Health Literacy (3 items) Interactive Health Literacy (3 items) Critical Health Literacy (5 items) For psychosocial health, six items measured ‘sense of certainty’ and four items assessed ‘life balance’. Additionally, semi-structured face-to-face interviews with a caregiver subgroup underwent content analysis.	Caregivers’ health literacy and psychosocial health	Three indices of caregivers’ health literacy: Functional HL (knowledge); Interactive HL (capability to act); Critical HL (individual empowerment).	**Individual Outcomes:** Caregivers showed significant improvements in Functional Health Literacy (knowledge), Interactive Health Literacy (capability to act), and Critical Health Literacy (empowerment). Improvements in functional (p=0.000) and interactive (p=0.000) health literacy were statistically significant. Additionally, caregivers reported better psychosocial health. They demonstrated enhanced stroke-specific knowledge and capability to act, with professionals noting increased individual empowerment. **System-Level Outcomes:** From the professionals’ perspective, the program influenced their routines, inter-institutional support, patient care quality, and cooperation. It also increased their awareness of the complexity of caregivers’ needs.
**Heckel et al., 2018^(16)^**	RCT	PROTECT	Zarit Burden Interview; Centre of Epidemiologic Studies - Depression scale (CES-D); Supportive Care Needs Survey for Partners & Caregivers (SCNS-P&C); Supportive Care Needs Survey (SCNS-SF34); Health Literacy Questionnaire (HLQ); Health education impact Questionnaire (heiQ); Self-esteem subscale of the Caregiver Reaction Assessment (CRA); Self-designed utility assessment.	Caregivers’ outcomes: caregiver burden; unmet needs; self-empowerment and health literacy; self-esteem; depressive symptoms; caregiver risk for depression. Patients’ outcomes: depressive symptoms, unmet needs; health literacy.	HLS Health literacy: S1 - feeling understood and supported by healthcare providers; S2 - having sufficient information to manage my health; S3 - actively managing my health; S4 - social support for health; S5 - appraisal of health information; S6 - ability to actively engage with health care providers; S7 - navigating the health care system; S8 - ability to find good health information; S9 - understanding health information well enough to know what to do.	**Caregiver outcomes:** Caregiver burden: no effect was observed after intervention p= 0.921; Caregiver unmet: reduction after intervention in both groups(IG baseline, mean = 2.66, 95% , CI [1.91-3.54]; group 1 month post intervention, mean = 0.85, 95%CI [0.42-1.44]; control group baseline, mean = 1.30 95%CI [0.80-1.94]; control group 1 month post intervention, mean = 1.02 95%CI [0.52-1.69]; p = 0.023); **Caregiver self-esteem:** decline in both groups from baseline to months 1 and 6 (p= 0.045). No significant difference between the groups over the time (p= 0.320). **Caregivers risk for depression:** caregivers at risk had a significant effect on having sufficient information to manage their health (p = 0.040); patients’ depressive symptoms, unmet needs; self-empowerment and health literacy levels no significant effects were found. **Patient outcomes:** No significant differences between groups on patients’ depressive symptoms, unmet needs and health literacy
**Ferré-Grau et al., 2013^(11)^**	RCT	ATDOM (home care program) + PST (problem-solving technique)	Questionnaire ‘Ad-Hoc’ (socio-demographic characteristics and care); Goldberg Scale - anxiety and depression in family caregivers.	Caregivers’ outcomes: Depression and anxiety.	Not reported	**Caregiver outcomes:** Statistically significant improvement in symptom of anxiety (p<0.05) and depression (p<0.01) after intervention.

### Interventions or programs on Mental Health

The studies included described six different interventions, namely PROTEC^(16)^, TIVA App^(15)^, and ATDOM (home care program) with addition to problem-solving technique^(11)^; Web-based psychoeducation course^(20)^, Family-centered empowerment model -FCEM^(18)^, and Caregivers-to-Caregivers Training Programme -C2C^(17)^, and Care-givers’Guide^(19)^.

The Interventions included home visits and problem-solving techniques^(11)^, smartphone app-based intervention program^(15)^, phone calls^(16)^, a Web-based psychoeducation course^(20)^, face-to-face sessions^(18)^, videoconferences, a chat group to discuss difficulties, and “personal support” when necessary^(17)^.

Regarding the number of sessions, they ranged from three^(16)^ to twelve^(17)^. Only one study indicated that the number of sessions was tailored as needed throughout the caregiving process^(19)^.

The strategies used in these sessions were diverse and included video conferencing, chat groups for discussing difficulties and “personal support” when necessary^(17)^; face-to-face sessions^(18)^; phone calls in the intervention group^(16)^; web-based psychoeducation courses^(20)^; daily activities from Monday to Friday based on the Positive Mental Health Decalogue^(15)^; sessions during usual home visits^(11)^ and through outreach counselling^(19)^. The duration of each session varied between 22 minutes^(16)^ and two hours and 30 minutes^(17)^.

Most interventions were conducted by nurses, except two studies that involved other professionals as a clinical social worker^(19)^ and a professional training group including psychologists, psychological counsellors, trained caregiver staff, and trained caregiver volunteers^(17)^. The outcomes of the studies primarily focused on caregivers, although one study also reported on patients’ outcomes^(16)^.

### Effectiveness and Outcomes

Caregivers’ outcomes included: health literacy, psychosocial health in caregivers, perceived preparedness of individual stroke caregivers^(19)^; depression and anxiety^(11,16)^; caregiver burden and positive mental health^(15)^; unmet needs, self-empowerment, self-esteem, caregiver risk of depression; caregivers’ knowledge, context-specific skills, and self-care ability^(17)^; care burden^(18)^; and engagement in the course^(20)^. Patients’ outcomes included depressive symptoms, health literacy, and unmet needs^(16)^. One study also reported outcomes on the system level^(19)^.

The effectiveness of the interventions varied, though improvements were reported in different outcomes. The study by Jiang et al.^(17)^ reported significant improvements in caregivers’ knowledge and skills from baseline to a two-month follow-up (p < .001). The same study showed that post-test and two-month follow-up scores were higher than baseline (p < .001), with improvements in understanding mental health conditions, recovery resources, coping strategies, patient symptoms and behaviours, communication skills, and patient advocacy.

Other results included significant differences between the intervention and control groups on the Positive Mental Health Scale (p = 0.001), specifically in problem-solving, self-realization (p < 0.001), pro-social attitude (p < 0.001), and self-control (p = 0.03)^(11)^.

Caregiver burden was assessed in two studies^(15,18)^. While one study found no significant effect of the intervention on reducing caregiver burden among caregivers of patients with multiple sclerosis^(18)^, another study reported a decrease in caregiver burden in the intervention group^(15)^. This study also showed that caregivers at risk of depression had a significant increase in having enough information to manage their health (p = 0.040), although self-empowerment and health literacy levels did not show significant effects. Another included study reported improvements in caregivers’ health literacy across all three indices: Functional HL (knowledge), Interactive HL (capability to act), and Critical HL (individual empowerment)^(19)^. These improvements were statistically significant in Functional HL (p = 0.000) and Interactive HL (p = 0.000). An increase in caregivers’ psychosocial health was also reported. Through qualitative methodology, caregivers observed improvements in their stroke-specific knowledge (functional health literacy) and capability to act (interactive health literacy). Professionals perceived improvements in caregivers’ individual empowerment (critical health literacy).

The study by Heckel et al.^(16)^ reported improvements in unmet caregiver needs following the ‘PROTECT’ intervention in both the control and intervention groups. Significant improvements were observed in symptoms of anxiety (p < 0.05) and depression (p < 0.01) after the intervention, which included problem-solving techniques and home visits.

Regarding patients’ outcomes, the intervention did not result in significant differences in health literacy and its domains^(16)^. One study also described the program’s outcomes at the system level from the professionals’ perspective^(19)^. Professionals reported that the new program influenced their professional routines, inter-institutional support, the quality of patient care, and inter-institutional cooperation. Additionally, it was noted that the program increased professionals’ awareness of the complexity of caregivers’ needs. More detailed information is available in Chart 2.

## DISCUSSION

Informal, unpaid care is essential in supporting health and long-term care systems. It is estimated that if an additional 10% of older people currently receiving informal support were to rely on formal care, public spending on long-term care in EU countries would double^(21)^.

The literature underlines the importance of health literacy and the mental health of informal caregivers as essential determinants for the quality of care they provide, particularly in the context of long-term care for adults and older adults with chronic pathologies. On the other hand, positive mental health literacy emerges as an actual relevant concept to the caregivers as it highlights the importance of nurturing the competence in problem-solving and self-actualization; personal satisfaction; autonomy, relatedness and interpersonal relationship skills, self-control, and prosocial attitude^(9)^. In this sense, this systematic review aimed to identify interventions designed to improving the mental health of informal caregivers, with an emphasis promoting health literacy and the analyzing of the health gains resulting from it.

The main results highlight the importance of interventions targeted at caregivers, underlining their impact on promoting the well-being of this population^(11,15,19)^. Educational and psychosocial interventions emerge as indispensable tools, contributing to increasing knowledge and improving skills. Interventions focused on health literacy effectively empower caregivers, promoting their empowerment and positively reflecting on psychosocial well-being and reducing burden, determining factors for quality of life and the sustainability of care provided.

Other studies corroborate these results, although several authors highlight the results heterogeneity, making it difficult to formulate generalizable conclusions about the effectiveness of the interventions^(22)^. For example, the results of Krieger et al.^(19)^ reinforce the effectiveness of educational interventions in promoting health literacy, highlighted as a modifiable factor capable of reducing health disparities^(23)^. These authors identified an association between low levels of health literacy in caregivers and less effective self-management behaviours on the part of care, greater use of health services and increased burden. Promoting health literacy down to the critical level not only improves the health of caregivers, but also that of those who depend on them, developing skills that go beyond basic knowledge, strengthening the ability to make informed decisions, self-efficacy, and resilience^(24)^.

The empowerment of caregivers is also reflected in the recognition of the importance of self-care as a central element for their positive mental health and well-being^(11,15,19)^. This aspect is particularly relevant in disadvantaged socio-economic contexts or in situations involving pathologies of high emotional and physical impact^(19)^.

However, the literature points to significant heterogeneity in interventions and outcomes aimed at informal caregivers^(22)^. While some psychosocial interventions are effective in reducing burden and promoting positive mental health, as in the studies of Ferre-Grau et al.^(11,15)^, others demonstrate inconclusive results or no significant impact^(18)^. Similarly, educational strategies vary in their outcomes: while face-to-face interventions are widely described as effective, digital tools emerge as more convenient and sustainable alternatives^(17,18,19)^. An example of this is the study by Ferre-Grau et al^.(15)^, which evaluated the use of a mobile application to support caregivers, demonstrating effectiveness in reducing the long-term burden and improving positive mental health, especially, in the factors associated with Teresa Lluck’s Multifactorial Model of Positive Mental Health: (F5) Problem solving and self-actualization, (F2) Prosocial attitude, and (F3) Self-control^(12)^. However, the success of these technologies depends on the digital literacy of caregivers, as well as access to devices and connectivity^(25,26)^.

Another aspect highlighted is the importance of sharing experiences between caregivers, either in peer groups or through community agents, promoting support networks that reduce social isolation and strengthen emotional support^(18,19)^. Additionally, strategies such as problem-solving techniques and home visits have been associated with reduced anxiety and depression^(11,16)^.

The diversity of strategies reflects the concern of health professionals, particularly nurses, to adapt interventions to the specific needs of caregivers and the contexts in which they are inserted^(22)^. The same source reinforces the relevance of multidisciplinary teams to ensure continuity and effectiveness at different levels of care. Nurses stand out as the main intervenors due to their proximity to caregivers and the central role they play in motivating and implementing change^(15,18)^.

While educational interventions prove to be effective, their personalization of caregivers’ needs and contexts is crucial, including cultural adaptation, as demonstrated by Ramos et al.^(27)^. However, significant gaps persist in the assessment of direct outcomes on caregiver well-being, pointing to the need for further research and long-term follow-up^(16,28)^.

In conclusion, it is essential that nurses and researchers objectively assess the needs of caregivers, developing interventions that integrate care and empowerment partnerships. This effort is vital to identifying caregivers in greater vulnerability, offering adequate support, and mitigating the adverse impacts of intensive and long-term care provision^(22)^. Investing in the personalization of interventions and recognizing the cultural and social specificities of caregivers is a necessary path to ensure the overall well-being of caregivers and the people in their care^(29,30)^.

### Study limitations

Despite the evidence of efficacy, the interventions analyzed have vary in approaches and duration. While some studies have used multiple face-to-face sessions, such as the study by Jiang et al.,^(17)^, others have adopted less frequent approaches such as occasional phone calls. The heterogeneity of interventions makes it difficult to directly compare the results of the studies, compromising the generalization of the conclusions.

The absence of two of the 13 quality assessment criteria (criteria five and six), inscribed in the results of the JBI critical evaluation checklist in the seven studies analyzed is understood as a limitation, suggesting the persistence of investment in methodological rigour in future research.

The scarcity of robust long-term studies is also considered a significant limitation, which makes it difficult to measure the real impact of mental health interventions on caregivers. In addition, some interventions have shown less expressive results in reducing caregiver burden in specific conditions, such as multiple sclerosis^(18)^, which indicates that caregivers’ needs may be even more complex and specific than initially anticipated. On the other hand, the diversity of interventions reveals a concern in adapting them to the specific needs of caregivers.

### Contributions to the Area

This review allows us to identify some implications relevant to practice, namely, that interventions are culturally sensitive, flexible and adaptable to the specific needs of caregivers and the health conditions faced.

The adoption of a multidisciplinary approach can enhance the benefits of interventions, providing holistic support, continuous support to caregivers and using strategies that combine educational and emotional support. Health literacy interventions have a potential positive effect on the quality of care provided to patients by improving the mental health and well-being of caregivers.

Future research should invest in randomized controlled clinical trials, ensuring methodological rigour, with the aim of evaluating the efficacy and long-term impact of interventions. It is also recommended to explore how health literacy can be optimized, considering the specificity of caregivers’ conditions (depending on the chronic disease responsible for care dependence), as well as the diversity of cultural contexts.

## CONCLUSIONS

This study shows that educational and psychosocial approaches, personalized and adjusted to the individual and cultural needs of caregivers, contribute positively to the reduction of burden, improvement of psychological well-being and the strengthening of self-management and resilience skills. It was shown that interventions aimed at improving the mental health of informal caregivers resulted in significant gains, namely those that promote health literacy and empowerment.

The intervention strategies are diversified, and a multidisciplinary approach is suggested, in order to ensure the continuity and effectiveness of care, although most of the interventions analyzed were led by nurses.

It is noteworthy that the development of interventions that combine educational, emotional and practical support has the potential to positively impact the mental health and well-being of caregivers, as well as reflects on the quality of care offered.

Although methodological limitations are identified and the heterogeneity of the studies prevents the generalization of conclusions, the results obtained suggest the relevance of investing in adjusted, flexible and culturally sensitive interventions to the needs and contexts of caregivers.

It is understood that promoting the mental health and well-being of caregivers is a strategy to support health systems, as well as a priority in health policies as a guarantee of the sustainability of care in the long term.

## Data Availability

The research data are available in a repository: https://doi.org/10.48331/scielodata.T5BEJK
